# Fear of Being Laughed at in Borderline Personality Disorder

**DOI:** 10.3389/fpsyg.2018.00004

**Published:** 2018-01-23

**Authors:** Carolin Brück, Stephanie Derstroff, Dirk Wildgruber

**Affiliations:** Department of Psychiatry and Psychotherapy, University Medical Center, Eberhard Karls University of Tübingen, Tübingen, Germany

**Keywords:** borderline personality disorder, gelotophobia, social cognition, laughter, fear of being laughed at

## Abstract

Building on the assumption of a possible link between biases in social information processing frequently associated with borderline personality disorder (BPD) and the occurrence of gelotophobia (i.e., a fear of being laughed at), the present study aimed at evaluating the prevalence rate of gelotophobia among BPD patients. Using the Geloph<15> , a questionnaire that allows a standardized assessment of the presence and severity of gelotophobia symptoms, rates of gelotophobia were assessed in a group of 30 female BPD patients and compared to data gathered in clinical and non-clinical reference groups. Results indicate a high prevalence of gelotophobia among BPD patients with 87% of BPD patients meeting the Geloph<15> criterion for being classified as gelotophobic. Compared to other clinical and non-clinical reference groups, the rate of gelotophobia among BPD patients appears to be remarkably high, far exceeding the numbers reported for other groups in the literature to date, with 30% of BPD patients reaching extreme levels, 37% pronounced levels, and 20% slight levels of gelotophobia.

## Introduction

Aside a pervasive pattern of instability in affect regulation, self-image, and impulse control, the list of traits that conceptualize borderline personality disorder (BPD) includes disturbances in interpersonal functioning as core clinical feature of the diagnosis (American Psychiatric Association, 2000; Lieb et al., 2004). In an effort to understand the underlying causes of BPD-related interpersonal dysfunction, research in recent years has devoted increasing attention to impairments in social cognition, i.e., impairments in the “mental processes involved in perceiving, attending to, remembering, thinking about, and making senses of the people in our social world” (Moskowitz, 2005, p. 3), as one likely contributor to BPD-related difficulties in social interaction. In this context, particularly failures to understand the emotions and intentions of others have frequently been the focus of research (Domes et al., 2008, 2009; Daros et al., 2013). Findings derived from this line of research lead to the assumption of a negativity bias in the evaluation of others (e.g., Arntz and Veen, 2001; Barnow et al., 2009; Domes et al., 2009) that drives patients to misperceive or misinterpret the nature of a social exchange possibly linked to a heightened “rejection sensitivity” (Miano et al., 2013), a “disposition to anxiously expect, readily perceive and intensely react to rejection” (Downey et al., 2004, p. 668) in BPD patients (Staebler et al., 2011).

Much like a lens through which BPD patients perceive the world, expectations of rejection may cloud patients’ interpretations of social interactions in a way that leads them to almost automatically perceive signs of rejections in others and to interpret perhaps even innocent or friendly interactions as rejecting (Downey et al., 2004).

At the level of observable behavior this processing disposition may reflect itself in a variety of forms – one possibly being the experience of *gelotophobia*, a fear of being laughed at (Ruch, 2009), based on the misinterpretation of laughter signals generally as signs of hostility and rejection. Studies of groups of people taken form the general population have contributed to the concept of gelotophobia as a continuum (Ruch et al., 2014), whereas particularly the high end of the range described as fear of being shamed by the ridicule of others with a paranoid sensitivity to anticipate ridicule, a disproportional negative response to laughter (Ruch et al., 2014), difficulties in regulating emotional states and an anger proneness (Weiss et al., 2012) may provide compelling suggestions of an overlap with BPD and BPD-related concepts such as rejection sensitivity. Though at first glance similar to personality dimensions such as shame-proneness or social anxiety, gelotophobia has been shown to transcend global personality traits and has been established as a unique concept distinct from more general traits used to describe an adult personality (Ruch et al., 2014). As far as links between social impairments in BPD and the concept of gelotophobia are concerned, behavioral studies conducted in groups of gelotophobic individuals once more draw attention to commonalities in responding. Similar to reports of a negative perception bias in BPD (e.g., Brück et al., 2017) studies show that gelotophobic individuals tend to perceive benevolent laughter as more unpleasant, misinterpret the affective state of a laughing individual as negative in valence and judge cartoons depicting social scenes involving laughter as displays of mockery and ridicule (Ruch, 2009).

Given the aforementioned phenomenological similarities between social cognitive impairments in BPD and markers of gelotophobia, one might assume a high prevalence of gelotophobics among BPD patients. While research conducted in groups of patients suffering from other mental disorders such as anxiety disorders, eating disorders, mood disorders, schizophrenic disorder or autism, confirm relatively high rates of gelotophobia among the studied patient groups (Forabosco et al., 2009; Samson et al., 2011), rates of gelotophobia among BPD patients remain unknown.

Bridging the current gap in research, this study aimed at evaluating the prevalence rate of gelotophobia among BPD patients. To this end, a group of BPD patients was asked to complete the Geloph<15> (Ruch and Proyer, 2008), a questionnaire that allows a standardized assessment of the presence and severity of gelotophobia symptoms.

## Materials and Methods

### Participants

A total number of 30 female patients (*M*_age_ = 23.47 years, age range: 19–34 years, *M*_education_ = 11.23 years ± 1.72 *SD*) diagnosed with BPD volunteered to participate. Participants were chosen from a pool of patients seeking treatment. Patients had to be 18 years or older and diagnosed with BPD in order to participate. Patients were diagnosed by trained psychiatrists or clinical psychologists based on the criteria provided in the Diagnostic and Statistical Manual of Mental Disorders (DSM-IV; Saß et al., 1996) using the International Personality Disorder Examination (IPDE; Loranger et al., 1998).

In addition to the diagnosis BPD, 15 of the 30 recruited BPD patients (=50%) also met the diagnostic criteria for mood disorders, nine (=30%) for eating disorders, nine (=30%) for anxiety disorders, five for disturbances of activity and attention (=17%), and 17 (= 57%) for substance abuse disorders. Seventeen of the 30 patients received some form of psychiatric medication: Six patients were treated with antidepressants, one with antipsychotics, ten with a combination of antidepressants and either antipsychotics, anxiolytics or mood stabilizers.

### Materials

To quantify symptoms of gelotophobia, each participant was provided with a German version of the Geloph<15>, a standard instrument to determine the presence and intensity of the fear of being laughed at (Ruch and Proyer, 2008). Measures of gelotophobia are obtained using a set of 15 statements describing typical behaviors and attitudes of gelotophobes. Participants are asked to indicate the extent of their agreement with each statement choosing one of four answer alternatives: strongly disagree, moderately disagree, moderately agree, and strongly agree.

To determine individual gelotophobia scores for each participant, ratings are assigned numeric values ranging from 1 to 4 with higher values indicating a higher degree of agreement with each statement (1 = strongly disagree, 2 = disagree, 3 = agree, 4 = strongly agree). The respective rating values then are averaged among all 15 items of the questionnaire resulting in a total score ranging from 0 to 4. Interpretation guidelines and cutoff values provided by the authors allow further detailing the findings: Averaged scores of 3.5 or higher are generally interpreted to represent expressions of extreme gelotophobia, while scores falling between the limits of ≥3.0 and <3.5 are interpreted to indicate a marked/pronounced gelotophobia, and scores between the limits of ≥2.5 and <3.0 a slight form of gelotophobia. Averaged scores lower than 2.5 are interpreted to indicate that the respective individual experiences no fear of being laughed at. Based on these cutoff values and their individual scores patients were categorized into one of four groups – patients without gelotophobia, with slight gelotophobia, with pronounced gelotophobia, and with extreme gelotophobia – and the percentage of BPD patients falling within each group was determined.

### Procedure

After admission to an inpatient-treatment program targeting individuals suffering from BPD, patients were approached by the first or second author and informed about ongoing studies concerning BPD and asked for their participation. Participation was voluntarily and did not interfere with the treatment program. Patients were given time to consider their participation and were revisited a few days after the first meeting. If a patient agreed to participate, a date at the patient’s earliest convenience was scheduled to collect the data. Data were collected in a quiet room separate from the ward patients sought treatment at. Participants were seated at a desk, handed a paper version of the Geloph<15> and asked to answer each questions without any constraints on the time needed to fill in the questionnaire. All participants had the opportunity to withdraw consent at any time during the study. Participants did not receive immediate feedback on their results. However, if a person asked for feedback, the results were explained.

### Data Analysis

For a more in-depth interpretation of the gathered data, findings on the relative frequency with which the different severity levels of gelotophobia occur in BPD patients were compared to prevalence rates obtained in other clinical and non-clinical reference groups. As far as the non-clinical reference group is concerned, a sample of 30 female volunteers was recruited from the general population. The selection of individuals was based on the criteria of a similar age (*M*_age_ = 23.93 years, age range: 18–31 years) and level of primary education (*M*_education_ = 11.47 years ± 1.57 *SD*) as BPD patients included in this study as well as on the criterion of not having been diagnosed with a psychiatric disorder. Prevalence rates for clinical groups were derived from research reports published in the current literature (Forabosco et al., 2009; Samson et al., 2011).

Chi-square tests were used to statistically compare proportions of gelotophobes and non-gelotophobes within the different groups, and odds ratios were calculated to estimate effect sizes (=odds of gelotophobia in BPD group (number of BPD patients with gelotophobia divided by number of BPD patients without gelotophobia) divided by odds of gelotophobia in reference group (number of individuals in reference group with gelotophobia divided by number of individuals in reference group without gelotophobia).

## Results

BPD patients’ Geloph<15> scores ranged from 2.00 to 3.93. Across patients, the scores averaged to a mean of *M*_Geloph_ = 3.13 (*SD* = 0.53). Categorizations based on cutoff values provided by Ruch and Proyer (2008) revealed that 26 out of the 30 BPD patients included in this study (=86.67%) could be classified as gelotophobic (i.e., Geloph<15> score > 2.5). 30.00% of all patients (=9/30) indicated extreme levels of gelotophobia, 36.67% (=11/30) pronounced levels, and 20.00% (=6/30) slight levels.

Comparisons conducted with prevalence rates in other clinical or non-clinical reference group (**Table 1**) indicated significantly higher rates of gelotophobia among BPD patients as relative to females without mental disorders [*χ*^2^(1, *N* = 60) = 38.57, *p* < 0.01 two-tailed] or patient with autism [*χ*^2^(1, *N* = 70) = 12.75, *p* < 0.01 two-tailed], schizophrenia [*χ*^2^(1, *N* = 56) = 8.86, *p* < 0.01 two-tailed], or mood disorders [*χ*^2^(1, *N* = 62) = 28.60, *p* < 0.01 two-tailed]. Based on odd ratios calculated on the data, the odds of BPD patients exhibiting gelotophobia were 91.0 times higher than in females without mental disorders, 7.9 times higher than in patients diagnosed with autism spectrum disorder, 6.5 times higher than in patients diagnosed with a schizophrenia spectrum disorder, and 28.2 times higher than in patients diagnosed with mood disorders.

**Table 1:** Percentages of individuals with no, slight, pronounced, and extreme gelotophobia summarized for patients with borderline personality disorders as well as non-clinical and clinical reference groups.

	*N*	*NG*	*G*_total_	*G*_s_	*G*_p_	*G*_e_
Borderline personality disorder	30	13.33%	86.67%	20.00%	36.67%	30.00%
Non-clinical reference group	30	93.33%	6.67%	6.67%	0.00%	0.00%
Clinical reference group						
Autism spectrum disorders^1^	40	55.00%	45.00%	27.50%	10.00%	7.50%
Schizophrenia^2^	26	50.00%	50.00%	15.38%	30.77%	3.85%
Mood disorders^2^	32	81.25%	18.75%	12.50%	3.12%	3.12%


*NG, no gelotophobia; Gtotal, gelotophobia (regardless of severity); Gs, slight gelotophobia; Gp, pronounced geloto-phobia; Ge, extreme gelotophobia. ^1^Data provided in Samson et al. (2011), ^2^Data provided in Forabosco et al. (2009)*.

## Discussion

In sum, the data gathered in this study evidence a high prevalence of gelotophobia among BPD patients: Roughly 9 out of 10 patients meet the Geloph<15> criterion for being classified as gelotophobic with more than 60% of all BPD patients exhibiting pronounced to extreme levels of symptom manifestation. Relative to occurrences in other clinical and non-clinical samples, the rates of gelotophobia among BPD patients appears to be rather high, far exceeding the numbers reported for other mental disorders (see **Table 1**) in the literature to date. Keeping in mind limitations of a medium-sized all female sample of patients currently seeking intensive psychotherapy, interpretations concerning gelotophobic traits in BPD patients (male and female) within the general population must remain cautious at this time. Particularly when considering data suggesting responses of female BPD patients on self-report measures to be clouded by a more negative world view, a greater dissatisfaction and critical views of themselves as compared to male BPD patients (McCormick et al., 2007), further studies with a mixed-sex sample become necessary to substantiate the current observation.

Besides a phenomenological description, at this point of time further studies should aim to advance our understanding as to what contributes to such extraordinarily high prevalence rate of gelotophobia and to detail how co-occurrences of gelotophobia may affect or even further complicate social lives of BPD patients.

As suggested earlier, specific cognitive-affective dispositions in the processing of social information – particularly the overreaching expectation of rejection – may provide an initial stepping stone to explain the occurrence of gelotophobia in BPD patients. Latter assumption builds on the idea that instead of correctly inferring different communicative intentions, the expectation of rejection leads patients to perceive laughter generally as sign of rejection and that coupled with the emotional turmoil of feeling rejected laughter becomes a signal to be feared – a misjudgment with perhaps dire consequences on a patients well-being and life in the community (e.g., social withdrawal, low self-esteem and social competence, or a lack of liveliness, spontaneity, and joy; Ruch, 2009). In this context, attention needs to be devoted to studying whether or not BPD patients indeed show impairments in the decoding of laughter signals in order to further substantiate the initial claims. While the hypothesis may be in line with reports of a reduced ability to correctly derive social information from other communication signals such as facial expressions (Domes et al., 2009; Daros et al., 2013), for example, to our knowledge no study to date has sought to investigate BPD-related alterations in the perception of laughter. Behavioral profiles derived from such studies combined with measures of gelotophobia and rejection sensitivity in the same samples of patients ultimately may allow to test the suggested model in which gelotophobia is mediated by specific dispositions of information processing and thus serves as another marker of a biased social perception in BPD.

With respect to the field of BPD treatment, knowledge about BPD-related phenomena such as gelotophobia may aid treatment planning, in a sense, that it may suggest to raise awareness to gelotophobic tendencies and their effects on social perception and to further discrimination learning with respect to social signals which ultimately may facilitate the cognitive restructuring of negative schemas regarding social interaction associated with BPD.

## Ethics Statement

The study was performed according to the principles of the Code of Ethics of the World Medical Association (Declaration of Helsinki) and with the approval of the ethics review board of the University of Tübingen. Before inclusion in the study, all participants gave written informed consent.

## Author Contributions

CB contributed to the conception and design of the study, to acquisition of participants, collection, analysis, and interpretation of data, as well as manuscript drafting and revision. SD contributed to the acquisition of participants, as well as the collection, analysis and interpretation of the data. DW contributed to the conception and design of the study, to analysis, and interpretation of data, as well as manuscript drafting and revision.

## Conflict of Interest Statement

The authors declare that the research was conducted in the absence of any commercial or financial relationships that could be construed as a potential conflict of interest.
